# Description of the consumption of toxics in patients with assertive community treatment and prolonged release treatment

**DOI:** 10.1192/j.eurpsy.2021.2182

**Published:** 2021-08-13

**Authors:** L. Garcia, C. Rodriguez, A.I. Willems

**Affiliations:** 1 Csm Eria, SESPA, Oviedo, Spain; 2 Csm La Calzada, SESPA, Gijon, Spain; 3 Csm Cangas Del Narcea, SESPA, Cangas del Narcea, Spain

**Keywords:** severe mental disorder, ACT, drugs, schizophrénia

## Abstract

**Introduction:**

The Assertive Community Treatment (ACT) was developed by Leonard Stein and Mary Ann. The objective is the treatment of serious Mental Disorders in an integral way and in the community.

**Objectives:**

The Assertive Community Treatment (ACT) was developed by Leonard Stein and Mary Ann. The objective is the treatment of serious Mental Disorders in an integral way and in the community.

**Methods:**

This is a retrospective study with a total of 69 patients whose main diagnosis is Schizophrenia undergoing CT follow-up in 2018-2019. The data obtained have been analyzed by the SPSS statistical program.

**Results:**

Our sample is mainly composed of men (60.9%) with an average age of 48 years (+ - 11.56). The main diagnosis is schizophrenia (62.3%) and the most commonly used long-term injectable treatment is paliperidone palmitate with a dose range of 150mg. Of the total number of patients, 29% of the cases did not maintain active use of any toxic, and the most commonly used toxic is tobacco (49.3% of cases).

**Conclusions:**

The inclusion of patients in a ACT program requires a diagnosis of severe Mental Disorder and poor therapeutic adherence. After analyzing our data, we observed that most of them also have active toxic consumption and high doses of psychotropic drugs.

**Disclosure:**

No significant relationships.

